# Cholera toxin — A foe & a friend

**Published:** 2011-02

**Authors:** Joaquín Sánchez, Jan Holmgren

**Affiliations:** Facultad de Medicina, UAEM, Av. Universidad, Col. Chamilpa, Mexico; * *University of Gothenburg Vaccine Research Institute (GUVAX) & Department of Microbiology & Immunology, The Sahlgrenska Academy at University of Gothenburg, Gothenburg, Sweden*

**Keywords:** Allergy, autoimmune disease, cholera toxin phage, cholera vaccine, GM1 ganglioside, immunological tolerance, intracellular toxin traffic, receptor binding site, toxin secretion, virulence gene regulation

## Abstract

After De’s pivotal demonstration in 1959 of a diarrhoeogenic exo-enterotoxin in cell-free culture filtrates from *Vibrio cholerae* (of classical biotype), much insight has been gained about cholera toxin (CT), which is arguably now the best known of all microbial toxins. The subunit structure and function of CT, its receptor (the GM1 ganglioside), and its effects on the cyclic AMP system and on intestinal secretion were defined in the 1970s, and the essential aspects of the genetic organization in the 1980s. Recent findings have generated additional perspectives. The 3D-crystal structure of CT has been established, the CT-encoding operon has been shown to be carried by a non-lytic bacteriophage, and in depth knowledge has been gained on how the bacterium controls CT gene expression in response to cell density and various environmental signals. The mode of entry into target cells and the intracellular transport of CT are becoming clearer. CT has become the prototype enterotoxin and a widely used tool for elucidating important aspects of cell biology and physiology, *e.g*., cell membrane receptors, the cyclic AMP system, G proteins, as well as normal and pathological ion transport mechanisms. In immunology, CT has emerged as a potent, widely used experimental adjuvant, and the strong oral-mucosal immunogenicity of the non-toxic B-subunit (CTB) has led to the use of CTB as a protective antigen together with killed vibrios in a widely licensed oral cholera vaccine. CTB has also been shown to promote immunological tolerance against certain types of mucosally co-administered antigens, preferably tissue antigens linked to the CTB molecule; this has stimulated research and development to use CTB in this context for treatment of autoimmune and allergic diseases. In summary, in the 50 years after De’s discovery of CT, this molecule has emerged from being the cholera patient’s “foe” to also becoming a highly useful scientist’s “friend”.

## Cholera toxin structure

Koch in 1884 proposed that the symptoms caused by *Vibrio cholerae* could be due to a “poison”. However, it was not until 1959 when the existence of such a cholera toxin (CT) was conclusively demonstrated. S.N. De, then in his now classical one-page Nature paper^1^, could report that cell-free culture filtrates from *V. cholerae* (of classical biotype) when instilled directly into ligated loops of the small intestine of rabbits could induce intestinal fluid accumulation. In 1969 Finkelstein and LoSpalluto^2^ had purified the toxin and shown it to be a 84 kDa protein. The toxin was initially thought to consist of only one type of subunit that could form aggregates of various sizes and presumed different toxicity. However, this picture was rapidly modified when Lönnroth and Holmgren^3^ and others^4^ demonstrated that CT is made up of two types of subunits, a 56kDa oligomer composed of several identical “light” subunits responsible for receptor binding and a single “heavy” 28kDa toxic-active subunit; these subunits were later renamed B (for binding) and A (for toxic-active), respectively. Simultaneously, the cell membrane receptor for CT was identified to be a specific ganglioside, GM1, which was arguably the first ever chemically fully defined biologic receptor^4^,^5^.

**Fig. 1:**
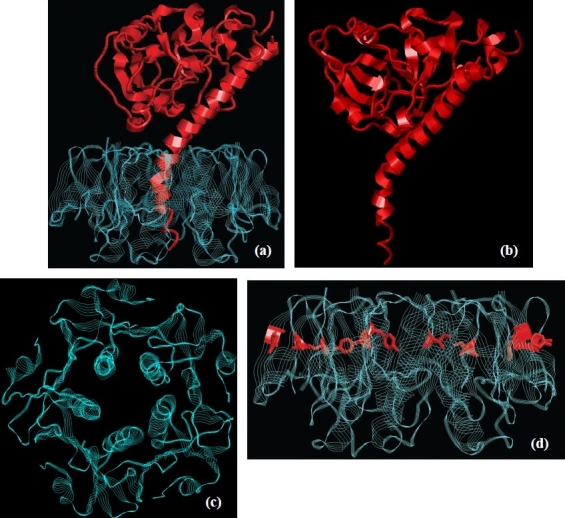
Crystallographic structure of cholera toxin **(a)**, its A **(b)** and B-subunits **(c)**. In **(d)** the position of the residues in CTB differing between pre-1993 El Tor and Classical CTs are highlighted.

Further studies defining the primary structure of CT and also its 3-D structure by high-resolution electron microscopy and crystallography have confirmed and extended these findings^6^,^7^. In the assembled CT (Fig. 1a) the toxic-active A-subunit (CTA, Fig. 1b) is embedded in the circular B-subunit homopentamer (CTB pentamer, Fig. 1c) responsible for toxin binding to cells. The 28 kDa CTA comprises 240 amino acids, and the 11.6 kDa B subunit monomers each has 103 amino acids. Although being synthesized as a single polypeptide chain, CTA is post-translationally modified through the action of a *V. cholerae* protease that generates two fragments, CTA1 and CTA2, which however still remain linked by a disulphide bond. The toxic (enzymatic ADP-ribosylating) activity of CTA resides in CTA1, whereas CTA2 serves to insert CTA into the CTB pentamer.

The CTB pentamer is held together by approximately 130 hydrogen bonds and 20 salt bridges. These many polar bonds together with a tight packing of subunits via hydrophobic interactions could by themselves explain the outstanding stability of pentameric CTB to proteases, bile components and other factors in the intestinal milieu. It has been suggested that pentamer-pentamer interactions may possibly further add to the stability.

The interactions between the CTB pentamer and CTA (specifically CTA2) are non-covalent and the last four amino acids (lysine-aspartate-glutamate-leucine; KDEL) at the carboxy-terminal of CTA2 protrude from the associated toxin and are not engaged in interactions with the pentamer. Taking the crystal structure of the heat-labile toxin (LT) from *Escherichia coli* as a reference^6^, many of the amino acid residues in the CTB pentamer that point towards the interior of the pore are charged, some being charged negatively and others positively. Charge neutralization calculations leave an excess of positive charges inside the pore, and some of these “free” positive charges in the CTB pentamer pore are supposed to interact with negatively charged residues in CTA2.

There are more than 140 *V. cholerae* serogroups and among them only a few may produce CT and cause disease. The overwhelming majority of clinical cases have been found to be due to infection by organisms belonging to serogroup O1 or, more recently, also serogroup O139, although other serogroups may cause sporadic cholera outbreaks. Based on biological properties, members of serogroup O1 can be further sub-divided into the so-called Classical and El Tor biotypes; the O139 vibrios are derivatives of the O1 El Tor organism. Traditionally the El Tor (and O139) and classical biotypes were considered to differ in the type of CT produced^8^. Although the A-subunits of the El Tor and Classical CT are identical in amino acid sequence, the B-subunits have been shown to have biotype-specific amino acid substitutions at positions 18 and 47^9^. Tyr-18 and Ile-47 were considered typical of the El Tor (and O139) biotype, and His-18 and Thr-47 typical of the Classical biotype. However, recently there appears to have been a striking change in clinical isolates of *V. cholerae* more or less world-wide, such that current O1 El Tor strains (as well as current clinical isolates of O139) have been found to produce CT of the classical biotype with His-18 and Thr-47 in CTB^10^. Actually, variability in both the type of CT (CTB) and the phage where the CT genetic information resides has been comprehensively analyzed^11^. In Fig. 1d, the CTB positions 18 and 47 have been highlighted to show that both amino acids have their side chains exposed; these residues can be presumed to be part of the epitopes that determine the specificity of biotype-specific anti-cholera toxin monoclonal antibodies. It should be noted that these residues do not take part in binding to GM1 and thus they are unlikely to influence affinity for the receptor. This would agree with the known indistinguishable toxic activity of CT isolated from previously typical El Tor and Classical *V. cholera*^12^.

## Genetics and regulation of cholera toxin

The structural genes encoding A and B subunits, *ctxA* and *ctxB*, are arranged so that the *ctxA* cistron precedes *ctxB*^13^. These CT genes are carried by a prophage designated CTXΦ that forms filamentous non-lytic particles^14^. CTXΦ may be a special kind of filamentous phage because besides being able to produce viral particles it can either integrate into the *V. cholerae* chromosome(s) or, in contrast to other filamentous phages, replicate as a plasmid. The genetic components and the insertion position of CTX
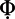
in either of the two *V. cholerae* chromosomes may vary.

The CTXΦ genome consists of a core region (4.5 kb) and an RS2 region (2.4 kb). The core region encodes CT and proteins that are required for viral morphogenesis, while the RS2 region encodes the regulation (RstR), replication (RstA), and integration (RstB) functions of the CTXΦ genome. The CTXΦ prophage is often flanked by a genetic element known as RS1. When RS1, RS2 and the core region of El Tor and Classical *V. cholerae* strains are compared one finds that their sequences are not identical. Therefore, in general El Tor and Classical strains carry biotype-specific CTXΦ phages, which are discerned through the sequence of RstR^15^; however, recent El Tor strains have been shown to carry CTXΦ prophage of the Classical type^11^. Interestingly, in O1 El Tor, and also in O139 *V. cholerae*, other forms of ctxAB transmission by a different *V. cholerae* filamentous phage, designated VGJ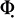 have been proposed^16^. The VGJΦ phage is reported to use the El Tor-specific mannose-sensitive haemagglutinin (MSHA) pilus as a receptor.

Similar to other bacteriophages, CTX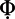 requires a receptor on *V. cholerae* for attachment and transmission. This receptor has been identified as the toxin co-regulated pilus (TCP), a pilus of approximately 8 nm in diameter and 1-4 mm in length, which is composed of some 1000 interwoven TcpA subunits^14^. The tcp operon encoding TCP resides separately from the CTXΦ in the large *V. cholerae* chromosome in the so-called Vibrio Pathogenicity Island (VPI).

**Fig. 2:**
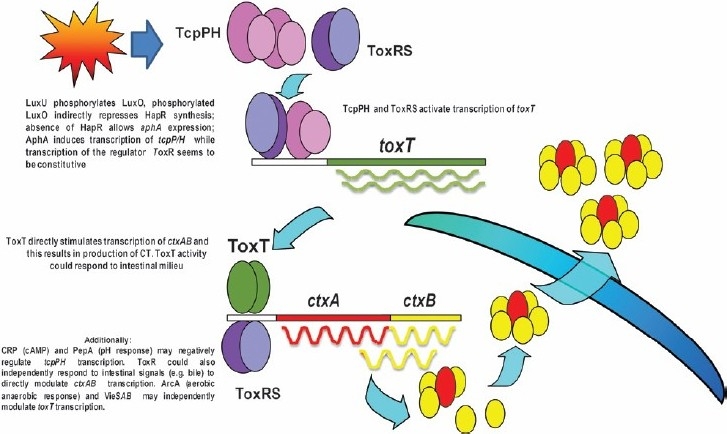
Diagrammatic representation of cholera toxin gene regulation.

Recent work in many laboratories has given much insight in the complex regulation of CT expression in *V. cholerae* (Fig. 2). Notably, VPI codes for a regulatory protein, ToxT, which can directly activate transcription of both the *ctxAB* (Fig. 2) and the tcp operons in a co-ordinated manner^17^. The expression of ToxT in *V. cholerae* is tightly regulated. The membrane-localized transcriptional activators ToxR/ToxS and TcpP/TcpH are required to activate transcription of the *tox*T gene by binding to a region upstream of the *toxT* promoter. The role of TcpH, the companion of TcpP, is to prevent the degradation of TcpP^18^. Part of the regulation mediated by ToxT depends on the regulator ToxR and this protein is encoded in the large chromosome outside the VPI and CTXΦ. Like TcpP, ToxR also exists as a complex with a companion protein, ToxS, but ToxS might operate different to TcpH and serve only to stabilize ToxR in an active, dimeric form.

Expression of CT is subject to regulation by several transcriptional activators controlled by the *V. cholerae* “quorum-sensing system”^19^. At low cell densities expression of CT increases, while at high cell density the quorum sensing system downregulates CT synthesis. At low cell density, there is phosphorylation of the regulator LuxO by LuxU (Fig. 2). Phosphorylated LuxO, via an indirect mechanism that involves degradation of the *hapR* mRNA encoding the HapR protein, increases the concentration of the positive regulator AphA, and the presence of this positive regulator leads to expression of other positive regulators TcpP and TcpH that activate expression of ToxT (Fig. 2). Increased levels of the ToxT regulator strongly stimulate transcription of *ctxAB* with the concomitant production of CT (Fig. 2). Besides TcpP and TcpH, activation of *toxT* requires both of the positive regulators ToxR and ToxS (Fig. 2), but expression of these latter proteins seems to be constitutive. At high cell densities the converse situation occurs; LuxO is dephosphorylated, the *hapR* message accumulates and HapR is produced. The HapR protein then represses *aphA* and the absence of the positive regulator AphA leads to decreases in TcpP, TcpH and ToxT and as a consequence CT expression is reduced. Apart from this regulatory pathway, the regulators ToxR and ToxS might, independently of ToxT, directly activate expression of *ctxAB* (Fig. 2).

In addition to the quorum sensing system, several other signals may affect the expression of CT in the intestinal milieu. Both cAMP and pH can modulate the activity of ToxT via inhibitory effects on *tcp*PH transcription, and ArcA (aerobic anaerobic response) and VieSAB may independently modulate *toxT* transcription. Further, ToxR has also been found to respond to intestinal signals such as bile to directly modulate *ctxAB* transcription^20^.

The regulatory systems described may allow *V. cholerae* bacteria to express CT (and TCP) in a tightly controlled spatio-temporal manner within the gut. This notion is supported both by *in vivo* experiments in mice^21^ and by the time-related transcriptional activation of *toxT* during *in vitro* culture^22^. Gene expression patterns in cholera stool-derived vibrios have shown that the *tcp* and *ctxAB* operons are largely repressed^23^. These results are consistent with earlier experiments that indicated repression of *ctxAB* expression upon growth of vibrios in sterile cholera stool fluid^24^.

## Cholera toxin secretion

CT is secreted by *V. cholerae* and is transported extracellularly by the so-called type II secretion system. This system comprises more than a dozen interacting proteins and serves to export toxin and other proteins such as extracellular enzymes from the periplasm across the outer membrane^25^. In *V. cholerae* the CT secretion system (named Eps, for extracellular protein secretion) contains pseudopilins that may form a pilus to extrude substrates to the extracellular space via a pore in the outer membrane (EpsD) using a mechanism analogous to a piston. Energy for secretion likely comes from EpsE, a cytoplasmic ATPase. The activity of these secretory proteins may be diverse, for example, the EpsD secretin from *V. cholerae* is required both for type II secretion and for extrusion of the CTXΦ^26^.

CT is secreted from *V. cholerae* after its assembly in the periplasm^27^,^28^. Although pentameric CTB can be secreted even in the absence of CTA, there appears to be a mechanism to normally ensure exit of fully assembled toxin (and no wasteful secretion of empty B pentamers). Thus *in vivo* the formation of B pentamers was found to be aided by the presence of cholera toxin A-subunits^28^, suggesting that the A-subunit may act as a nucleation center for holotoxin assembly in the periplasm. Enhanced assembly mediated by the A-subunit would favour secretion of holotoxin. Moreover, A-subunits in the absence of CTB are not transported across the *V. cholerae* external membrane^27^. Unincorporated A-subunits could be prone to degradation, in analogy to protein hybrids derived from LTA in *E. coli*^29^.

## Mode of action of cholera toxin – from cell binding to diarrhoea

CT is released from *V. cholerae* cells in a very efficient manner, and more than 90 per cent of the toxin is usually found extracellularly in a soluble form^27^. Once in the intestinal lumen, CT initiates its toxic action on cells by binding with high affinity and exquisite specificity to cell membrane receptors, which were identified more than 30 years ago as the monosialoganglioside GM1: [Gal(β1-3)GalNac(β1-4)(NeuAc(α2-3)Gal(β1-4)Glc]→ ceramide^4^,^5^. GM1 is present in many cell types, and CT can be demonstrated to bind to (and intoxicate) different types of cells experimentally. However, in non-synchronized cultures not all exposed cells will bind and internalize CT because GM1 expression on the cell surface is a cell cycle-dependent process with preferential binding in G_0_/G_1_^30^.

**Fig. 3:**
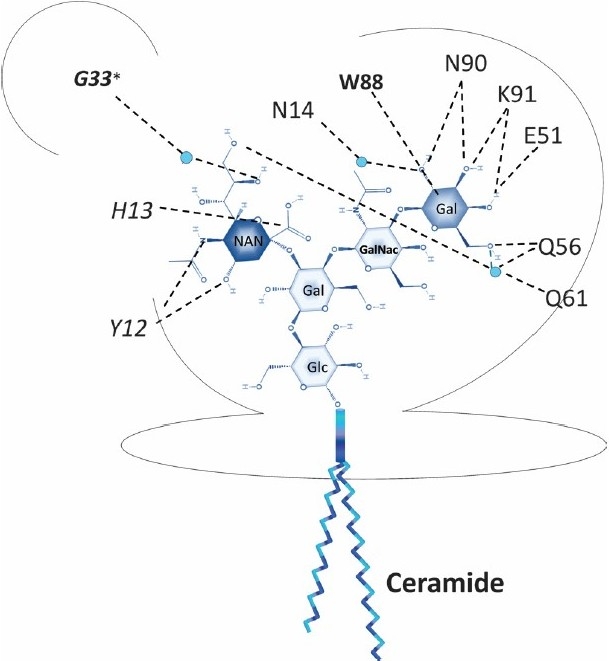
Cholera toxin B-subunit GM1 receptor binding pocket The pentasaccharide structure of the GM1 receptor is shown (Gal, stands for Galactose, GalNac for N-acetylglucosamine; NAN for N-acetylneuraminic acid and Glc for Glucose).The Gal and NAN sugar residues in green and red are those that establish direct interactions with the B-subunit, either directly or via the solvent (small spheres) Interactions, mostly hydrogen bonds, are depicted by broken lines. The amino acid residues of CTB found to be indispensable for binding are in bold and larger font. The asterisk specifically denotes the amino acid residue (Gly33) that comes from the adjacent B subunit. All indicated interactions involve side chains of amino acids except for those shown in italics. (Lengths of broken lines are not meant to depict real atomic distances; also, relative locations of amino acids are merely diagrammatic).

Both the specific sugar residues in GM1 and the amino acid residues in CTB interacting with each other have been defined and based on published data^31^,^32^ we diagrammatically represent those interactions (Fig. 3). Although there is one GM1-binding site in each B subunit monomer, a single amino acid (Gly33* in Fig. 3) from the neighbouring CTB monomer also has a role in the binding^31^, explaining the dramatically higher binding strength of the CTB pentamer compared with that of individual B subunit monomers. Critical residues for interaction with GM1 binding have been defined as Trp88, Gly33 (from adjacent monomer) and Tyr12.

**Fig. 4:**
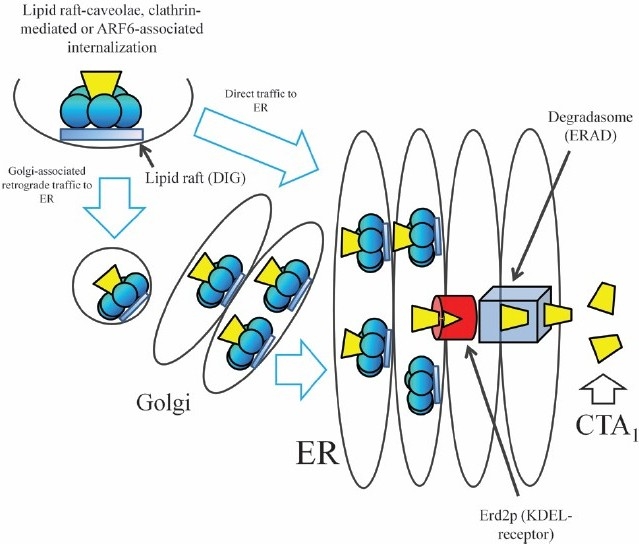
Cholera toxin intracellular traffic.

After binding to GM1, which appears to be localized mainly in lipid rafts on the cell surface, CT is endocytosed by the cell. In order for cell intoxication to occur, the A subunit (or, more specifically, CTA1) needs to be transported to the cytosol to induce the activity of adenylate cyclase (AC). A schematic summary for intracellular toxin transport is presented in Fig. 4. The precise mode by which CTA1 reaches the cytosol is still not fully resolved. However, CT or pentameric CTB may be endocytosed, depending on cell type, either through caveolin-coated vesicles, clathrin-coated vesicles or by the so-called Arf6 endocytic pathway. After endocytosis, CT or the CTB pentamer travels to the endoplasmic reticulum (ER) via a retrograde transport pathway which, possibly dependent on cell type, may be Golgi dependent or independent^33^. There is association of the CT-GM1 complex with the actin cytoskeleton via lipid rafts and it is thought that the actin cytoskeleton has a role in CT trafficking from the plasma membrane to the Golgi-ER^34^.

After CT has reached the ER, CTA dissociates from CTB. The KDEL carboxy-terminal of CTA2 is a classical eukaryotic signal for retention in the ER lumen. This sequence was initially thought to be crucial for localization of the whole toxin to the ER, but it is more probable that the KDEL sequence serves to enhance retrieval of dissociated CTA from the Golgi apparatus to the ER, instead of being essential for retrograde transport^35^. Similar to the endocytosis and retrograde transport of CT to the ER, the translocation of CTA1 to the cytosol also engages a natural recycling cellular process, the so-called ER-associated degradation or degradasome pathway, which normally retrieves misfolded proteins from the ER for their degradation in the cytosol^36^. However, although being transported by the degradasome, CTA1 apparently escapes proteolysis, presumably because of its low content of lysine, which is the target for ubiquitinylation. To pass through the degradasome, CTA1 would have to undergo unfolding and refolding, a process possibly involving reduction by protein disulfide isomerase (PDI) followed by reoxidation by Ero1^37^.

The entry of CTA1 to the cell cytosol is the key step for intoxication because CTA1 catalyzes the ADP ribosylation of the trimeric Gsα component of AC. This enzymatic reaction is allosterically activated by the so-called ADP-ribosylation factors (ARFs), a family of essential and ubiquitous G proteins. Crystal structures of a CTA1/ARF6-GTP complex reveal that binding of the ARF activator elicits striking changes in CTA1 loop regions that allow the nicotinamide adenine dinucleotide (NAD+) substrate to bind to the active site^38^.

After ADP-ribosylation by CT, the AC remains in its GTP-bound state resulting in enhanced AC activity and an increased intracellular cAMP concentration. Higher levels of cAMP produce an imbalance in electrolyte movement in the epithelial cell, namely a decrease in sodium uptake together with an increase in anion extrusion, mostly chloride, by the cystic fibrosis trans-membrane conductance regulator (CFTR)^39^. Decreased sodium uptake reduces water intake by the enterocyte, and, at the same time, augmented chloride and bicarbonate extrusion gives rise to sodium outflow, and thus water secretion. The combined effect produces vast fluid loss from the intestine, in extreme cases up to 2 liters per hour.

Besides the direct effect of CT on AC activity and cAMP production in enterocytes, it has been proposed that the diarrhoeal response to CT might have a significant neurological component. It is thought that CT stimulates enterochromaffin cells to release serotonin, and that serotonin then promotes the release of the secretagogue vasointestinal peptide from intestinal neural networks^40^.

## Cholera toxin and immunology

In recent years, the immunological properties of CT and LT have attracted a great deal of attention. Both CT and LT are exceptionally potent oral-mucosal immunogens and these have also been found to be strong adjuvants for many co-administered antigens. These properties may be explained by three main characteristics of the CT and LT molecule. First, consistent with their functions as potent enterotoxins, these proteins are remarkably stable to proteases, bile salts, and other compounds in the intestine. Secondly, as discussed above, both CT and LT also bind with high affinity via their B-subunits to GM1 ganglioside receptors, which are present on most mammalian cells including not only epithelial cells, such as the “M cells”covering the Peyer’s patches, but also all known antigen-presenting cells (APCs); this facilitates the uptake and presentation of the toxins to the gut mucosal immune system. Thirdly, CT and LT have strong inherent adjuvant and immunomodulating properties that depend both on their cell-binding and enzymic ADP ribosylating activities^41^.

*The CTB-whole cell oral cholera vaccine*: The toxicity of CT has precluded its use for human vaccination. Instead, nontoxic CTB has been extensively used without any side effects as a mucosal immunogen in humans. Indeed, recombinantly produced CTB is an important component of an oral cholera vaccine for human use. In addition to CTB, this vaccine also contains inactivated whole-cell cholera vibrios and is being registered (Dukoral®) in more than 60 countries worldwide^42^. The vaccine has proved to be safe and efficiently immunogenic in both adults and children. When given orally in two or three doses, the vaccine has been found to stimulate the same levels of intestinal IgA antitoxin and antibacterial (mainly anti-lipopolysaccharide) antibodies as seen in convalescents from severe form of clinical cholera. The vaccine has also been found to induce very long-lasting (more than 5 years) immunologic memory in the intestinal mucosa. Three large phase III field trials in Bangladesh, Peru and Mozambique have demonstrated a high protective efficacy of the vaccine, being 85-90 per cent for the first 6 months after vaccination in both endemic (Bangladesh and Mozambique) and non-endemic (Peru at the time of the study) populations^43^. In adults and children above age 5 yr, protection remains at or above 60 per cent for another 2-3 yr. In children below age 5, the vaccine induces very high short-term efficacy (100% for the first 4-6 months) mediated by locally produced IgA antitoxic antibodies, but in this age group protection waned more rapidly than in older children^43^. A large effectiveness trial undertaken in a high-endemic area of Mozambique showed that the oral CTB-whole cell cholera vaccine was safe and highly effective (80-90% protection) when used as a public health intervention tool in a population with a high frequency of HIV-infected individuals^43^. It may be concluded that antitoxic immunity contributes significantly to overall vaccine-induced immune protection in cholera for as long as there are specific antitoxin antibodies (approximately 9 months) whereas longer-term protection is due mainly to antibacterial antibodies produced locally in the gut as a result of infection-induced stimulation of IgA memory cells responding sufficiently rapidly to avert the infection before it causes symptoms.

Consistent with this explanation and because of the close immunological relationship between CTB and LTB, the CTB whole-cell cholera vaccine, in addition to protecting against cholera, has also been found in several placebo-controlled trials to provide 60-80 per cent short-term protection against diarrhoea caused by LT-producing *E. coli* causing cholera-like diarrhoeal disease (ETEC diarrhoea). ETEC diarrhoea is the most common bacterial enteric infection in most developing countries and is also a common illness affecting 20-30 per cent of all travelers to these countries^44^. Therefore, protection against ETEC diarrhoea mediated by the cross-reacting CTB component of the cholera vaccine is a significant extra benefit of cholera vaccination.

Based on its excellent safety and immunogenicity in humans when given by the oral route, the CTB-containing cholera vaccines as well as the isolated CTB component have often been used as model immunogens for studies of mucosal immune responses in humans after immunization through mucosa. Indeed, much of our current knowledge of the localization of the mucosal immune responses after different routes of immunization and of the links between mucosal inductive and expression sites in humans have emerged from studies in volunteers using CTB as immunogen^45^.

*CT and LT as mucosal adjuvants*: Besides being strong mucosal immunogens, both CT and LT are powerful mucosal adjuvants. They strongly potentiate the immunogenicity of most other antigens, whether these are linked to or simply admixed with the toxins, provided that the other antigen is given at the same time and at the same mucosal surface as the toxins^41^,^49^,^50^.

CT and LT can affect several steps in the induction of a mucosal immune response, which alone or in combination might explain their strong adjuvant action after oral immunization. Thus, CT has been found to (*i*) induce increased permeability of the intestinal epithelium leading to enhanced uptake of co-administered antigens; (*ii*) induce enhanced antigen presentation by various APCs; (*iii*) promote isotype differentiation in B cells leading to increased IgA formation; and (*iv*) exert complex stimulatory as well as inhibitory effects on T cell proliferation and cytokine production^41^. Additionally, both CT and LT have been shown not only to avoid the induction of oral tolerance but also to abrogate otherwise efficient regimens for tolerance induction achievable by oral antigen administration.

Among these many effects, those leading to enhanced antigen presentation by various APCs are probably of the greatest importance for the adjuvant activity. CT or LT markedly increase antigen presentation by dendritic cells, macrophages, and B cells^46^. These have also been found, at least *in vitro*, to stimulate intestinal epithelial cells to become effective APCs. Consistent with this activity, CT/LT upregulates the expression of MHC/HLA-DR molecules, CD80/B7.1 and CD86/B7.2 co-stimulatory molecules as well as chemokine receptors such as CCR7 and CXCR4 on both murine and human dendritic cells and other APCs. Importantly, CT/LT also induces the secretion of interleukin (IL)-1b from both dendritic cells and macrophages. IL-1 not only induces the maturation of dendritic cells, but also acts as an efficient mucosal adjuvant when co-administered with protein antigens and might mediate a significant part of the adjuvant activity of CT^47^.

To avoid the toxicity problems with whole CT or LT, the recombinantly produced CTB and LTB proteins have been explored for their ability to increase immune responses against co-administered antigens. However, their capacity as mucosal adjuvants has proven to be much less than that of the holotoxins. Indeed, both CTB and LTB are poor adjuvants when given to animals together with non-coupled antigens by the oral route, although they display a more significant adjuvant activity when administered via the nasal route^41^. Adjuvanticity of CTB or LTB is much improved when coupled to antigens. This is due both to the increased uptake of the coupled antigen across the mucosal barrier and to the more efficient GM1 receptor-mediated uptake and presentation of the coupled antigen by APCs including dendritic cells and macrophages as well as naïve B cells.

**Fig. 5:**
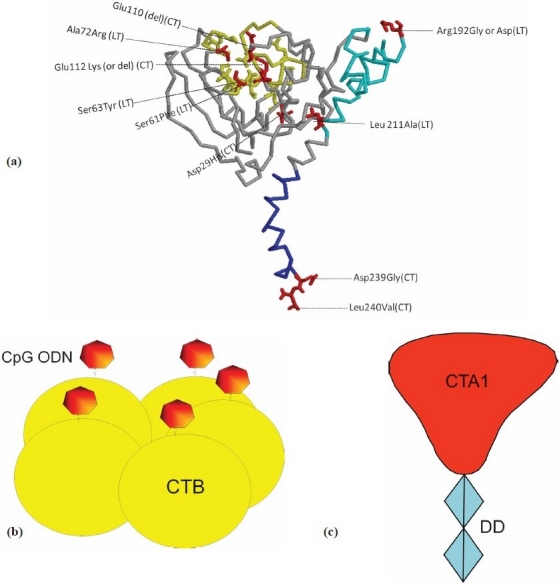
Adjuvant-active proteins based on CT and/or LT enterotoxins. **(a)** Amino acid changes and deletions in the A subunits described to result in adjuvant active toxins with no or much reduced enterotoxicity. The original amino acid is indicated, followed by its position along the mature A-subunit polypeptide sequence and then mutated amino acid. If the amino acid was deleted, this is indicated in parenthesis (*del*); the type of mutated toxin (CT or LT) is also shown in parenthesis. **(b)** A different type of adjuvant-active molecule based on a fusion protein between CTA1 and the immunoglobulin-binding protein DD (a derivative of *Staphylococcus aureus* protein A). **(c)** Adjuvant-active complex obtained by coupling TLR-9 activating CpG oligonucleotides (shown as heptagons) to CTB (coloured yellow) pentamers.

Recently, various molecular engineering approaches have permitted the generation of various LT and CT A-subunit mutants (Fig. 5a) some of which consist of polypeptide chain extensions^48^, that are substantially reduced in, or in some cases practically devoid of, enterotoxic activity, but retain detectable adjuvanticity when given to animals by a mucosal route^49^. The currently most promising of these mutant proteins appears to be the double-mutated LT with modifications in both residues 192 and 211 of the LTA subunit to inhibit toxin “nicking” and thus the enterotoxicity of the molecule; this molecule is currently scheduled for phase I clinical trials of its safety in humans when administrated in different dosages.

A different approach has been taken by Lycke^50^, who instead of attenuating the A-subunit made a gene fusion protein between fully active CTA1 and a *Staphylococcus aureus* protein-A derivative named DD (Fig. 5b). The CTA1-DD fusion protein binds specifically to immunoglobulins on antigen-presenting B cells via the DD protein and induces ADP ribosylation by the CTA1 moiety. When given intranasally together with other protein antigens, CTA1-DD substantially increases both mucosal and systemic immune responses. Yet another type of promising adjuvant protein was described by Adamson *et al*^51^. They coupled the well-known CpG oligonucleotide adjuvant (which activates innate immunity through interacting with toll-like receptor 9) to CTB (Fig. 5c) and showed that the CpG-CTB conjugate had markedly increased the activation of different APCs *in vitro* and thereby stimulating both T-cell and antibody responses *in vivo*.

*CTB for mucosal immunotherapy*: Mucosal tolerance is a mechanism whereby the immune system, upon encounter with harmless antigens through a mucosal surface, develops means to avoid reacting in a deleterious manner to the same antigen even if the antigen is encountered by a systemic route. This permits mammals to co-exist with their normal flora and to eat large amounts of foreign food proteins without inducing harmful systemic immune responses. Since induction of mucosal tolerance is antigen specific but can be expressed in a nonspecific manner (“bystander suppression”) via the production of suppressive cytokines by regulatory T cells in the inflamed microenvironment of the target organ, this approach has been utilized to suppress immune responses against self antigens. It has been possible to prevent or to delay onset of experimental autoimmune diseases in a variety of animal systems by feeding selected autoantigens or peptide derivatives^45^.

While mucosal tolerance is usually effective in animal models for preventing inducible autoimmune diseases, its efficacy has been more variable and limited when utilized as an intervention strategy in animals in which the disease had already been induced or had spontaneously developed. This may explain in part the disappointing results of recent clinical trials of oral tolerance in patients with type I diabetes, multiple sclerosis, and rheumatoid arthritis, diseases in which there may be multiple target autoantigens that remain largely unknown.

A significant improvement has been achieved by co-administering CTB as an immuno-modulating agent to enhance the tolerogenic activity of autoantigens as well as allergens given orally or nasally. The use of antigen coupled to CTB has been found to minimize by several hundred-fold the amount of antigen/tolerogen needed and also to reduce the number of doses that would otherwise be required by reported protocols of tolerance induction by the oral route^52^. More importantly, at divergence from the use of free antigen, CTB-linked antigens have been shown to work also in an already sensitized individual. As recently reviewed^53^, in experimental systems this has also resulted in effective suppression of various pathological immune responses associated with experimental autoimmune diseases, type I allergies and allograft rejection when the CTB-antigen conjugate was administered as therapy rather than for prevention. Recently, initial proof of principle of CTB-Ag-mediated tolerance in humans could be demonstrated. Thus, based on previous encouraging results in a rat model of heat-shock protein-induced uveitis, a small phase I/II trial in patients with Behcet’s disease (BD) was undertaken with very encouraging results^54^. BD is an autoimmune eye disease often associated with extraocular manifestations and abnormal T cell reactivity to a specific peptide (“BD peptide”) within the human 60 kD heat shock protein. Oral administration of CTB-BD peptide conjugate, three times weekly, had no adverse effects and enabled gradual withdrawal, without any relapse of uveitis, of existing treatment with immunosuppressive drugs in the majority of patients with BD.
